# Approximate Entropy of Brain Network in the Study of Hemispheric Differences

**DOI:** 10.3390/e22111220

**Published:** 2020-10-27

**Authors:** Francesca Alù, Francesca Miraglia, Alessandro Orticoni, Elda Judica, Maria Cotelli, Paolo Maria Rossini, Fabrizio Vecchio

**Affiliations:** 1 Brain Connectivity Laboratory, Department of Neuroscience & Neurorehabilitation, IRCCS San Raffaele Pisana, Via Val Cannuta, 247, 00166 Rome, Italy; francesca.alu@sanraffaele.it (F.A.); fra.miraglia@gmail.com (F.M.); alessandro.orticoni@sanraffaele.it (A.O.); paolomaria.rossini@policlinicogemelli.it (P.M.R.); 2 Department of Neurorehabilitation Sciences, Casa Cura Policlinico, 20144 Milano, Italy; e.judica@ccppdezza.it; 3 Neuropsychology Unit, IRCCS Istituto Centro San Giovanni di Dio Fatebenefratelli, 25125 Brescia, Italy; mcotelli@fatebenefratelli.eu

**Keywords:** entropy, EEG, left and right, brain networks

## Abstract

Human brain, a dynamic complex system, can be studied with different approaches, including linear and nonlinear ones. One of the nonlinear approaches widely used in electroencephalographic (EEG) analyses is the entropy, the measurement of disorder in a system. The present study investigates brain networks applying approximate entropy (ApEn) measure for assessing the hemispheric EEG differences; reproducibility and stability of ApEn data across separate recording sessions were evaluated. Twenty healthy adult volunteers were submitted to eyes-closed resting EEG recordings, for 80 recordings. Significant differences in the occipital region, with higher values of entropy in the left hemisphere than in the right one, show that the hemispheres become active with different intensities according to the performed function. Besides, the present methodology proved to be reproducible and stable, when carried out on relatively brief EEG epochs but also at a 1-week distance in a group of 36 subjects. Nonlinear approaches represent an interesting probe to study the dynamics of brain networks. ApEn technique might provide more insight into the pathophysiological processes underlying age-related brain disconnection as well as for monitoring the impact of pharmacological and rehabilitation treatments.

## 1. Introduction

Human brain, a dynamic complex system, can be studied using different approaches, including linear and nonlinear ones.

It has long been observed that human physiological parameters are nonstationary and nonlinear, and, in most circumstances, it is possible to detect only the macroscopic output of physiological functions. Electroencephalographic (EEG) signal represents an example of macroscopic output of brain activity; its analyses are based on different approaches such as the Fourier transform technique, which assumes linearity and stationarity of the EEG signal [1]; granger causality, which can be used to measure connectivity across brain regions [2,3,4,5]; and independent component analysis and general linear models (GLM) [6]. However, due to the complex and dynamical characteristics of brain systems and hemispheric differences, nonlinear approaches are expected to be more appropriate for exploring the physiological mechanisms of brain activity [1].

Hemispheric structural and functional differences have been well documented in healthy individuals. Several aspects make the brain structure asymmetrically distributed in the two hemispheres, such as white matter structure, gray matter volume, connections, and cortical thickness. These structural differences are assumed to be associated with hemispheric lateralization of functional specializations, including visuospatial processing, language, motor, and cognitive control [7].

EEG activity has been widely discussed in relation to functional neuronal mechanisms [8,9]. In this regard, it is of major interest to investigate how brain electric oscillations are synchronized in physiological or pathological brain states (e.g., in resting state or during an epileptic seizure) or by external and internal stimulation (event-related potentials (ERP) or evoked potentials (EP)). The study of the network of the cerebral hemispheres in the resting condition and related differences at the base is interesting as well. This matter can be investigated by applying nonlinear methods to the analysis of the EEG signals because changes in EEG activity even in resting-state conditions could be considered as a transition from a disordered to an ordered state (or vice versa) [10].

To assess hemispheric differences, several studies have used different methodological approaches, both from an instrumental point of view, using functional magnetic resonance imaging (fMRI) [11] and EEG [12], for instance, and concerning the parameter that has been used as an index of those differences. Indeed, data analysis can consist of graph theory approaches such as the small-world index [13,14], rich-club organization [15,16], and modularity [17,18].

Although linear approaches have shown the presence of a structured organization in brain networks [19], nonlinear approaches for resting EEG analysis may show differences in network organization.

For example, the entropy, defined as the measurement of predictability in any system, represents a nonlinear approach widely used in EEG analyses and is used to quantify the complexity of brain areas’ changes. Higher entropy values are usually associated with more random and less ordered systems.

The concept of entropy was introduced at the end of the 19th century by Clausius [20], the first one to enunciate the second law of thermodynamics by saying that “entropy always increases” [21]. Thus, the concept of entropy was initially conceived in the field of thermodynamics and statistical physics. During the same period, Boltzmann firstly enunciated the logarithmic connection between entropy and probability. Starting from 1948, Shannon [22,23] applied this concept to information theory and proposed a large number of applications in information science.

To explore the nonlinear dynamics of the physical systems, Kolmogorov entropy and Renyi entropy have been widely used [24,25]. To quantify the complexity of biological signals in the human system, such as heart rate, breathing, or EEG signals, different types of entropy measure can be applied, such as approximate entropy (ApEn) [26], sample entropy (SampEn) [27], and multiscale entropy (MSE) [28,29]. In the present study, we selected ApEn as the approach for the analysis, because of its good properties; in fact, this approach is one of the most used measures for entropy evaluation nowadays. In particular, ApEn is an index able to measure the regularity of sequences and time-series data and has been extensively used in studies of physiologic time series to assess the degree of randomness [30,31]. Several ApEn properties facilitate its usage: it can be applied with good reproducibility to time series of at least 50 samples; it is almost unaffected by noise; it is finite for composite, stochastic, and noisy deterministic processes [32]; and it detects the changes in underlying episodic behavior undetected by peak occurrences or amplitudes [33]. For all the exposed reasons, ApEn would be extremely helpful in brain function understanding, given the complex and dynamical characteristics of cerebral systems.

ApEn differs substantially from the entropy measure that most scientists have in mind, that is the Shannon entropy. ApEn measures the predictability of future amplitude values of time series based on the knowledge of the general one or two previous amplitude values; instead, Shannon entropy measures the predictability based on the probability distribution of amplitude values already observed in the signal [34]. Unlike the Shannon entropy, the calculation of ApEn is not predicated on the underlying distribution of the data; instead, it is based on sequence recurrence. This allows ApEn to be applied to signals of shorter length, and makes model estimation unnecessary, removing the risk for misestimating based on poor model selection [35].

Although entropy algorithms have been widely used in the analysis of EEG signals in different contexts [36,37,38], a very few studies have evaluated entropy applying ApEn measure to assess hemispheric differences in brain networks analysis.

For example, Hogan and colleagues [39] have examined entropy applied to EEG in young adults’ groups across different experimental conditions, in three brain regions (Frontal, Temporal, and Parietal) applying the sample entropy measure. Their results show entropy differences in young adults between the right and left hemisphere, in particular, higher entropy in the left than the right hemisphere in posterior brain areas.

Taking in mind all the mentioned and suggested studies and in order to verify and improve previous evidence, the present study investigates brain networks applying ApEn measure (an already implemented function available to all researchers) for assessing the hemispheric EEG differences, focusing on more regions (Frontal, Central, Parietal, Occipital, and Temporal), in particular, five regions instead of three, with respect to the previously mentioned study [39]. We also used 54 channels instead of 36; moreover, an additional aim of the present study was to evaluate the reproducibility and stability of ApEn measure over time across separate recording sessions at both a few minutes and 1-week distances.

## 2. Subjects and Methods

### 2.1. Participants

Twenty healthy volunteers were recruited for the present study (10 females and 10 males; mean age = 26.1 ± 0.7 (standard error) and mean education = 16.4 ± 0.4 (standard error)).

Exclusion criteria included a history of neurological or psychiatric disorder and current treatment with vasoactive or psychotropic medication. All subjects were right-handed on Handedness Questionnaire [40]. Subjects were submitted to eyes-closed resting EEG recording in four separate sessions of about 3 min each, for 80 recordings. The EEG sessions were structured as follow: between the first and second resting EEG sessions, there was a 1-min break; between the second and third sessions, there was a 1-h break; and between the third and fourth ones, there was a 1-min break.

Informed consent was obtained from each participant. Experimental procedures were conformed to the Declaration of Helsinki and national guidelines.

### 2.2. Data Recordings and Preprocessing

The four EEG recordings of each subject were carried out with the same digital EEG machine (BrainAmp by Brain Products) from 64 electrodes positioned according to the International 10–20 system. Two separate channels, vertical and horizontal EOGs, were used to monitor eyes blinking. Impedance was kept below 5 KΩ and the sampling rate frequency was set up at 1000 Hz. Electroencephalographic signals were measured at rest, in 3 min of eyes closed and no task condition. During the recording, subjects were sitting in a comfortable armchair, placed in a dimly lit, sound-damped, and electrically shielded room.

The data were processed in MATLAB (MathWorks, Natick, MA, USA) using scripts based on the EEGLAB toolbox (Swartz Center for Computational Neurosciences, La Jolla, CA, USA) [41].

The EEG recordings were downsampled at 512 Hz and band-pass filtered from 0.2 to 47 Hz using a finite impulse response (FIR) filter. Imported data were divided into 2 s duration epochs and principal artifacts in the EEG recordings (i.e., eye movements, cardiac activity, and scalp muscle contraction) were removed with Infomax ICA algorithm [42,43], which enables the separation of statistically independent sources from multichannel EEG recordings [44,45,46,47] as implemented in the EEGLAB. At the end of the artifact removal procedure, at least 2.5 min remained for each session. One subject was rejected because the recording was too noisy and showed a small number of artifact-free epochs (less than 2 min for all conditions).

### 2.3. Entropy Analysis

Entropy analysis was computed on the artifact-free epochs utilizing a homemade software developed in MATLAB. The core of the software is the ApEn evaluation, which was realized using the algorithm already implemented in MATLAB, i.e., approximateEntropy function estimates the approximate entropy of the uniformly sampled time-domain signal by reconstructing the phase space. The software is implemented as follows: for each channel and each epoch, a value of ApEn is computed; later, those values are averaged among the epochs, in order to obtain only one ApEn value for each channel and each EEG session.

Two input parameters, a pattern length, *m*, and tolerance factor, *r*, are specified to compute it. The ApEn generates a unitless number from 0 to 2, where an ApEn value equal to 0 corresponds to a perfectly regular time series, whereas an ApEn value equal to 2 is produced by random time series [48].

The ApEn is computed as follows [35]:

1. The first sequence of length *m* is compared to all the other sequences of the same length point by point. Those sequences for which all points are within *r* of their corresponding point in the original sequence are counted. *r* is also known as similarity criterion, and more clearly is a tuning parameter used to identify a meaningful range in which fluctuations in data are similar. So, a point of a sequence is similar to its corresponding point in the original sequence, when its value is not above its original value plus *r*.
2. The same process is applied to sequences of length *m* + 1, starting with the first sequence of *m* + 1 points.
3. The amount of similar sequences for *m* + 1 long one is divided by the one resulting from *m* long sequences comparison. The natural logarithm of the ratio is taken.
4. The process is repeated for all possible sequences.
5. All logarithms results are summed and normalized for *N*, the total number of data samples, and *m*.

Summarizing, the ApEn is calculated as $ApEn=\;\Phi_{m}-\Phi_{m+1}$, where

$$\Phi_{m}=\;{\left(N-m+1\right)}^{-1}\overset{N-m+1}{\underset{i=1}{\sum }}\log\left(N_{i}\right).$$

Moreover, $N_{i}$, the number of within range points, namely, the amount of points that are within *r* of their corresponding point in the original sequence, at the point *i*

$$N_{i}=\;\overset{N}{\underset{i=1,i\neq k}{\sum }}(\|Y_{i}-Y_{k}{\|}_{\infty }\;<r).$$

In the present study, the MATLAB default values for the input parameters were selected: so *m* was equal to 2 and *r* to 0.2 * variance (*x*) [35,49,50,51], where *x* is a 2 s long epoch of a specific channel.

These well-established values are selected because they have been demonstrated to produce good statistical reproducibility for time series of length *N* > 60 [26]. Normalizing *r* in this manner gives ApEn a translation and scale invariance; in this way, it remains unchanged under uniform process magnification, reduction, or constant shift to higher or lower values [32]. In the present study, since the sampling frequency was set to 512 Hz, time series of 2 s were 1024 points long [51].

Through the homemade software, ApEn was evaluated on every single epoch for each channel. Only later, the ApEn values were averaged over the epochs to get a single value of ApEn for each channel [52].

**Figure 1:**
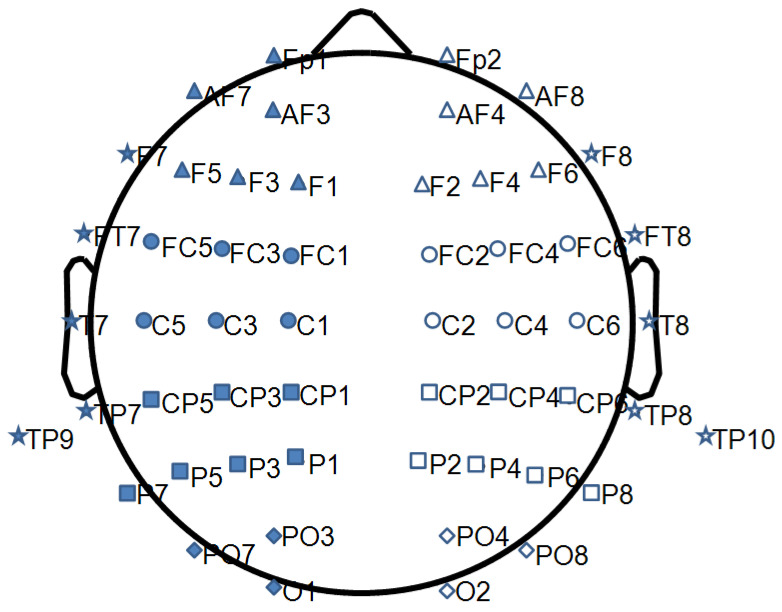
The figure shows the grouping of the electrodes for the regions of interest (ROIs of the brain networks) explored: full shapes for the left hemisphere and empty shapes for the right hemisphere. Triangle for frontal, circle for central, square for parietal, rhombus for occipital, and star for temporal ROI.

Once computed, in order to obtain a single value of entropy for each region of interest (ROI), the ApEn values evaluated in each electrode were averaged over ten ROIs (five left and five right: Frontal Left, Frontal Right, Central Left, Central Right, Parietal Left, Parietal Right, Occipital Left, Occipital Right, Temporal Left, Temporal Right), grouping the electrodes as shown in Table 1. The electrodes along the midline were excluded (Figure 1).

**Figure 2:**
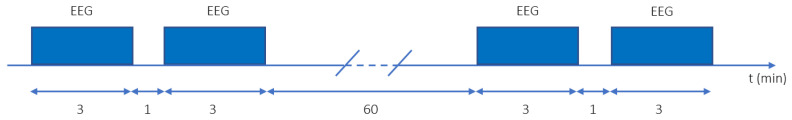
The diagram illustrates the schematic of the recording sessions. Each resting electroencephalography (EEG) session lasts 3 min; between the first and second session, there is a 1-min break; between the second and third session, there is a 1-h break; and between the third and fourth session, there is a 1-min break.

For greater clarity, we included a diagram illustrating the schematic of the recording sessions (Figure 2).

### 2.4. Statistical Evaluation

Analysis of variance (ANOVA) was used between the ROIs computed in the five regions for each hemisphere of the brain for all the recordings. Greenhouse and Geisser correction was used for the protection against the violation of the sphericity assumption in the repeated measure ANOVA. Besides, post hoc analysis with the Duncan’s test and significance level at 0.05 was performed.

A three-way ANOVA design was performed between the factors: Side (Left and Right), ROI (Frontal, Central, Parietal, Occipital, and Temporal), and Time (four sessions) with a statistical cutoff level of *p* < 0.05.

## 3. Results

The ANOVA for the evaluation of ApEn showed no statistically significant interaction (F (12, 288) = 0.30605, *p* = *0*.98808) among all factors, namely, Side (Left and Right), ROI (Frontal, Central, Parietal, Occipital, and Temporal), and Time (four sessions). More specifically, the ApEn analysis highlighted an optimal reproducibility of this measure in the sessions. Indeed, the statistical analyses showing that no interaction including Time resulted significant underlines the stability of the present methodology at least when carried out on relatively brief EEG epochs within a short time frame.

**Figure 3:**
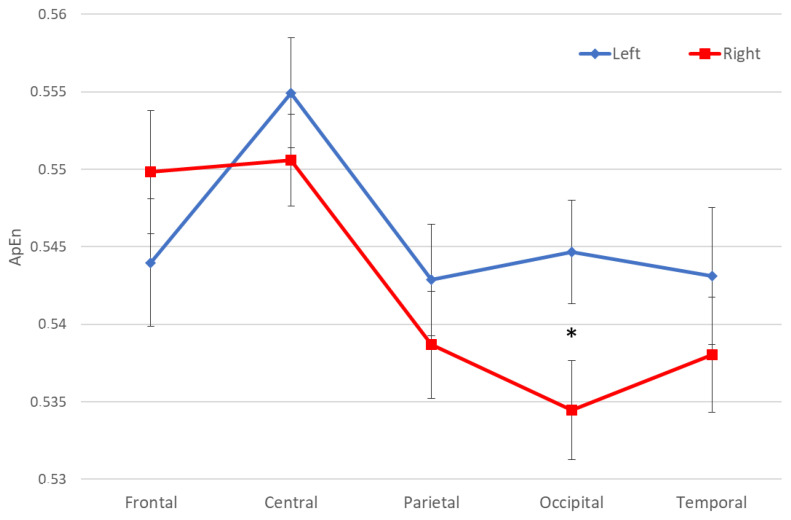
The figure shows the ANOVA analysis for the evaluation of entropy using the approximate entropy (ApEn) measure. For each region and each hemisphere, the ApEn values averaged across the population and the relative standard errors are reported. In the two hemispheres, there is a statistically significant interaction (F (4, 288) = 3.5825, *p* = *0*.00719) between the factors Side (Left and Right) and ROI (Frontal, Central, Parietal, Occipital, and Temporal). Post- hoc testing showed higher entropy values in occipital left compared to the right side (*p* < 0.001251), as highlighted by the asterisk.

Furthermore, the same ANOVA showed a statistically significant interaction (F (4, 288) = 3.5825, *p* = *0*.00719) only between the factors Side (Left and Right) and ROI (Frontal, Central, Parietal, Occipital, and Temporal), thus independently by the Time (Figure 3). The Duncan post hoc testing showed higher entropy values in the occipital left compared to the right side (*p* < 0.001251). It is also evident that higher values of entropy are represented almost exclusively in the left hemisphere, in fact, the main factor Side was also statistically significant (F (1, 72) = 4.4118, *p* = *0*.03919), showing a higher level of entropy in the left compared to the right hemisphere.

### Control Analyses

To evaluate the stability and reproducibility of the present measure, a control analysis was performed on a separate group of 36 young adults (18 females; mean age = 24.7 years, SD = 3.1). Subjects were submitted to resting EEG recording in two separate sessions of about 3 min each, for 72 recordings. Between the first and second resting EEG sessions, there was a 1-week break. The EEG recordings were acquired and analyzed with the same methodology as the main analyses.

The ANOVA for ApEn stability evaluation showed no statistically significant interaction (F (4, 140) = 0.21604, *p* = *0*.92916) among all factors: Side (Left and Right), ROI (Frontal, Central, Parietal, Occipital, and Temporal), and Time (session 1 and session 2), underlining the stability of the ApEn measure as evaluated with the present methodology even when performed within a longer time frame.

The shapes for the analysis of stability and reproducibility of the ApEn are not shown since the trend of the measure was the same represented in the main analysis (Figure 3).

Moreover, a comparative analysis aiming to test another measure of entropy to assess hemispheric differences was performed. In particular, the Shannon entropy has been chosen to evaluate if another measure could be able to detect the same or even more hemispherical differences than ApEn did. Shannon entropy has been largely employed not only in EEG analysis [34,53,54,55] but also in other fields, such as ECG one [56,57].

The comparative analysis was performed on the same group of subjects as the main analysis, thus on the same EEG recordings. The same ANOVA design of the main analysis was used.

The results showed a trend that is very close to that of ApEn. However, no statistically significant interaction (F (4, 68) = 2.0142, *p* = *0*.10222) has been revealed between the factors Side (Left and Right) and ROI (Frontal, Central, Parietal, Occipital, and Temporal). In particular, only a tendential trend (*p* = 0.1) has been found in the occipital region between the left and the right hemisphere, while the ApEn proved to be able to differentiate the two hemispheres in the mentioned area.

## 4. Discussion

The present study aimed to evaluate brain hemispheric differences using the nonlinear entropy property, evaluated specifically through the measurement of ApEn, applied to eyes-closed resting EEG recordings. Furthermore, we evaluated the reproducibility and stability of ApEn measure across separate recording sessions within a relatively brief period. From now on, we will refer to ApEn simply by entropy, without forgetting that entropy is a broad concept, while ApEn is the alternative, among the many ones, which has been chosen in the present study to measure entropy from data.

Entropy applied to EEG data has been demonstrated to clearly differentiate a normal awake brain state from a vegetative one and to represent a good predictor for recovery [58].

Our results showed significant differences in the occipital region, with higher values of entropy, measured by ApEn, in the left hemisphere than in the right one. Moreover, no significant interactions were found when the Time factor was included; this underlies the stability of the present methodology, at least when carried out in a short time lap.

The present finding in the occipital asymmetry is in line with previous evidence showing that entropy is higher in the left posterior regions than in the right ones [39].

We could speculate that this brain behavior stems from the different functions that the occipital areas of the two hemispheres support. Over the last decades, in effect, the brain interhemispheric differences have been widely explored to understand the neural basis of functional asymmetries and the factors that modulate cognitive specialization in the brain.

From a morphofunctional point of view, the brain can be seen as a set of partially independent neuronal systems, each dedicated to carrying out its specific function. However, the functional specificity of the cerebral neuronal systems does not imply their complete segregation, i.e., their activities are indeed coordinated by reciprocal links and by centers with diffuse projections that ensure the unity of brain activities [59].

In particular, the posterior regions of the brain receive information from the external world through the primary sensitive areas of parietal, occipital, and temporal lobes [60].

Besides, all the visual information is processed in the occipital lobe, including the information on posture and balance; indeed, one of the most important functions of the occipital lobe is receiving and interpreting visual information. Through the neurons of the visual areas, the occipital lobe is involved in functions such as reading, understanding of written language, visual perception, recognition of colors and shapes of objects, perception of depth, and recognition of moving objects. [61,62].

Several studies have explored the functions of the occipital lobe in different experimental conditions, showing how posterior brain regions of different hemispheres become active with different intensity also in closed-eyes conditions.

For instance, an fMRI study [63] exploring the differences in the hemisphere activation for two types of emotions, namely, basic emotions (e.g., anger and surprise) and self-conscious ones (e.g., pride and embarrassment) has found more activation for the latter than the former in left middle occipital regions in subjects inferring other people’s emotional states (decoding process).

Interestingly, injury to the occipital lobe appears to cause problems in object recognition such as in Anton syndrome, a disorder called cortical blindness where, while preserving intact peripheral environmental vision, subjects are unable to recognize objects [64].

A recent study has also found a relationship between the activity of the occipital regions and anxiety [65]. The left and right hemispheres of the brain are involved in different mechanisms for anxiety; indeed, the left hemisphere seems to be involved in its arousal. Moreover, it seems that the occipital cortex activity may reflect the anxiety state of healthy individuals.

Several studies have shown how the occipital areas of the two hemispheres are engaged differently in the cerebral functions [66,67]. Our data analysis showed a distribution of entropy that reflects how the hemispheres become active with different intensity. Entropy is a measure of signal predictability and quantifies the degree of the disorder of a system and the complexity of dynamic changes, so we could hypothesize that the high or low randomness values of the revealed electrical activity of a specific hemisphere are the result of the subject’s mental state and predisposition to the current activity. Both nonlinear and linear measures have certain advantages and disadvantages in the study of bioelectrical signals but due to the nonlinear and nonstationary properties of brain activity, nonlinear approaches to EEG analysis, such as entropy measurement, proved to be useful tools for researching physiological and pathological features of the brain networks [1].

The measurement of the ApEn represents an interesting probe to study brain networks. Thanks to the stability of this measure, further studies could evaluate its trend in the two hemispheres through different experimental conditions and different groups of subjects. Furthermore, in a clinical context, this technique might provide more insight into the pathophysiological processes underlying age-related brain disconnection as well as for monitoring the impact of eventual pharmacological and rehabilitation treatments.

## Figures and Tables

**Table 1:** The table shows the grouping of the electrodes for each region of interest (ROI) and for each hemisphere.

Brain Region	Electrodes						
Frontal Left	FP1	AF3	AF7	F1	F3	F5	
Frontal Right	FP2	AF4	AF8	F2	F4	F6	
Central Left	FC1	FC3	FC5	C1	C3	C5	
Central Right	FC2	FC4	FC6	C2	C4	C6	
Parietal Left	CP1	CP3	CP5	P1	P3	P5	P7
Parietal Right	CP2	CP4	CP6	P2	P4	P6	P8
Occipital Left	PO3	PO7	O1				
Occipital Right	PO4	PO8	O2				
Temporal Left	F7	FT7	T7	TP7	TP9		
Temporal Right	F8	FT8	T8	TP8	TP10		
Medial	FPz	Fz	Cz	CPz	Pz	POz	Oz

## Data Availability

The data that support the findings of this study are available on request from the corresponding author.
